# The expression levels of prolyl oligopeptidase responds not only to neuroinflammation but also to systemic inflammation upon liver failure in rat models and cirrhotic patients

**DOI:** 10.1186/s12974-015-0404-7

**Published:** 2015-09-30

**Authors:** Jofre Tenorio-Laranga, Carmina Montoliu, Amparo Urios, Vicente Hernandez-Rabaza, Hanan Ahabrach, J. Arturo García-Horsman, Vicente Felipo

**Affiliations:** Real-time Imaging Laboratory, Divisions of Pharmacology and Toxicology and Pharmaceutical Biosciences, Faculty of Pharmacy, University of Helsinki, Viikinkaari 5E, PO Box 56, Helsinki, 00014 Finland; Fundación Investigación Hospital Clínico Universitario, INCLIVA, Valencia, Spain; Laboratory of Neurobiology, Centro de Investigación Príncipe Felipe, Avd.Autopista del Saler 16, 46012 Valencia, Spain

**Keywords:** Prolyl oligopeptidase, Hepatic cirrhosis, Minimal hepatic encephalopathy, Liver failure

## Abstract

**Background:**

Liver failure in experimental animals or in human cirrhosis elicits neuroinflammation. Prolyl oligopeptidase (PREP) has been implicated in neuroinflammatory events in neurodegenerative diseases: PREP protein levels are increased in brain glial cells upon neuroinflammatory insults, but the circulating PREP activity levels are decreased in multiple sclerosis patients in a process probably mediated by bioactive peptides. In this work, we studied the variation of PREP levels upon liver failure and correlated it with several inflammatory markers to conclude on the relation of PREP with systemic and/or neuroinflammation.

**Methods:**

PREP enzymatic activity and protein levels measured with immunological techniques were determined in the brain and plasma of rats with portacaval shunt (PCS) and after treatment with ibuprofen. Those results were compared with the levels of PREP measured in plasma from cirrhotic patients with or without minimal hepatic encephalopathy (MHE). Levels of several pro-inflammatory cytokines and those of NO/cGMP homeostasis metabolites were measured in PCS rats and cirrhotic patients to conclude on the role of PREP in inflammation.

**Results:**

In PCA rats, we found that PREP levels are significantly increased in the hippocampus, striatum and cerebellum, that in the cerebellum the PREP increase was significantly found in the extracellular space and that the levels were restored to those measured in control rats after administration of an anti-inflammatory agent, ibuprofen. In cirrhotic patients, circulatory PREP activity was found to correlate to systemic and neuroinflammatory markers and had a negative correlation with the severity of the disease, although no clear relation to MHE.

**Conclusions:**

These results support the idea that PREP levels could be used as indicators of cirrhosis severity in humans, and using other markers, it might contribute to assessing the level of neuroinflammation in those patients. This work reports, for the first time, that PREP is secreted to the extracellular space in the cerebellum most probably due to glial activation and supports the role of the peptidase in the inflammatory response.

## Background

Hepatic encephalopathy (HE) is a complex neuropsychiatric syndrome resulting from chronic or acute liver disease; 30–50 % of patients with liver cirrhosis present minimal forms of HE [1]. Minimal HE (MHE) is the first stage in the spectrum of HE [2, 3], and it is usually diagnosed using the psychometric HE test [4]. Two main factors contribute to the neurological alterations in HE: hyperammonemia and inflammation [5–7]. In patients with minimal HE, cognitive impairment correlates with serum levels of inflammatory cytokines IL-6 and IL-18 [8]. We have shown that neuroinflammation is produced upon mimicking liver failure in rats [5, 6], which models human HE. Furthermore, anti-inflammatory drugs as ibuprofen [5] or MAPK-p38 inhibitors [9] have shown to restore cognitive function in PCS rats.

Prolyl oligopeptidase (PREP; EC 3.4.21.26) is a serine peptidase able to cleave short peptides (<30 amino acids) at the C-side of a proline residue [10, 11]. Although present in all organs, PREP is localized in specific cells and cell layers across the brain and peripheral tissues [12]. In the mature healthy brain, PREP is highly expressed in a certain group of neurons in well-defined areas such as the striatum, cortex, hippocampus and cerebellum but scarcely detectable in glial cells [13, 14]. PREP has been associated to neurodegeneration [10, 11], proliferation [15], neuronal differentiation [16], development [17, 18] and also to inflammation [19–21]. PREP levels have been found altered in Alzheimer’s (AD), Parkinson’s (PD) [22] and Huntington’s (HD) diseases (reviewed in [10]). In experiments on PREP overexpressed neuroblastoma cells, the data suggest that PREP has a role on secretion and clearance of α-synuclein deposits [23, 24]. Recent data also indicate that PREP is involved in hormonal regulation [25–27]. It has been shown that upon an inflammatory insult PREP expression is dramatically increased in astrocytes and microglia in the mouse brain [20]. Increased activated glia in AD and PD has been also related to PREP expression increase [22]. Previous studies demonstrate that under specific stimulus activated microglia can express and secrete PREP, which, in vitro, is toxic for neurons, effect that is partially prevented by specific inhibitors of PREP [28].

Results so far suggest that the function of PREP is different in different cell types and depends on the peptidase location: intercellular or extracellular [24]. Emerging evidence suggests that PREP participates in the inflammatory response through the modulation of active peptides [19, 21].

Although active research on PREP has been carried out recently, there is still lack of information on the specific physiological and pathological mechanisms where this peptidase is involved. Different reports indicate an involvement of PREP in several inflammatory diseases such as chronic obstructive pulmonary disease [29, 30], lupus erythematosus [31, 32], rheumatoid arthritis [33–35] and bronchiolitis obliterans syndrome [36] among others [20]. Recently, it has been reported that circulating PREP activity is significantly decreased in patients of multiple sclerosis (MS) [19, 37]. Although that the mechanistic link between PREP and MS has not been defined, it seems that it is related to inflammation in a complex fashion, where α-2-macroglobulin, fibrinogen and thymosin β-4 might have a role [19]. Furthermore, in experimental autoimmune encephalomyelitis (EAE), a mice model of MS, we observed that animals become more sensitive to the autoimmune challenge when they are administrated with PREP inhibitors, compared with vehicle-treated EAE mice [19].

In this work, we hypothesized a decreased of circulating levels of PREP in cirrhosis and wanted to find out if the relationship was linked to systemic inflammation or/and neuroinflammation, as judged by the development of HE. We also intended to find additional information on the possible role of PREP as a biomarker of (neuro) inflammation. Additionally, if there were PREP changes in cirrhosis and/or HE, we also wanted to find out if those were correlated to changes in inflammatory markers, like IL-6, IL-8, cGMP, atrial natriuretic peptide and nitrates/nitrites, in order to find a possible mechanistic link between the pathological states.

## Methods

### HE rat model of portacaval shunt (PSC)

Male Wistar rats were subjected to portacaval anastomosis as described by Lee and Fisher [38]. Control rats were sham operated. The experiments were approved by the ethical committee of the Centro de Investigación Principe Felipe complying with the European Community guidelines for experimental animal care and management.

### In vivo microdialysis

Rats were anesthetized with isoflurane, and a microdialysis cannula (CMA, Stockholm, Sweden) was implanted in the cerebellum (AP −10.2, ML −1.6 and DV −1.2), as described in [39]. After 48 h, a microdialysis probe (CMA/12; 3 mm long) was implanted in the freely moving rat and perfused (3 μl/min) with artificial cerebrospinal fluid (in mM): NaCl, 145; KCl, 3.0; CaCl2, 2.26; buffered at pH 7.4 with 2 mM phosphate. After a 2–3-h stabilization period, samples were collected every 30 min for 6 h. PREP activity was measured in samples as described below. Activity was determined by triplicate for each time point (see figure caption).

### Immunohistochemistry

PREP density was determined in xylene dewaxed brain sections, rehydrated with graded alcohols and washed with 0.1 M phosphate-buffered saline (PBS). The antigen retrieval was processed in a microwave oven in citrate buffer (pH 6.0) for 3 × 5 min. Endogenous peroxidase activity was inactivated with 5 % hydrogen peroxidase for 5 min and non-specific binding was blocked with 10 % Normal Rabbit Serum (Product# S-5000; Vector Laboratories) in PBS. Primary antibody (1:500, affinity purified chicken IgY, [14, 20, 40]) was added and incubated overnight at +4 °C in moist chambers. After two PBS washes, the sections were incubated with the secondary antibody (1:1000, rabbit anti-chicken FITC, #31501, Pierce Biotechnology, or 1:1000 anti-goat horseradish peroxidase complex, Santa Cruz Biotechnology Inc., Dallas, TX, USA) for 2 h. Double immunofluorescence with PREP antibody was performed after PBS washes. Vectashield (Product # H-1000, Vector Laboratories) or Vectashield with DAPI (Product # H-1200, Vector Laboratories) was used as mounting medium and to visualize the cell nuclei. Density was determined in digitalised images in at least three randomly selected fields per tissue on PCA or control sections, from 4 to 9 animals (see figure captions).

For PREP cellular localization experiments by confocal microscopy, brains were dissected out and postfixed in the same fixative and, subsequently, transferred to phosphate buffer (PB) with 0.1 sodium azide. Coronal sections (30 μm) were cut on a vibratome (Leica VT 1000S) and were stored at 4 °C in PB with 0.1 % azide until further processing. Free-floating sections were washed and sequential incubations with blocking serum and primary antibodies (overnight 4 °C) were performed. Brain sections were stained with anti-PREP (1:300, Abcam, UK), anti-GFAP (1:400, Sigma-Aldrich, USA) and anti-NeuN (1:100, Merck-Millipore, Germany). Finally, the secondary antibodies Alexa fluor 488 Donkey anti-mouse, Alexa fluor 555 Donkey anti-goat and Alexa fluor 647 Donkey anti-rabbit (all of them, 1:400, Invitrogen, USA) were incubated for 1 h at room temperature. To assess the co-expression of PREP with astrocytes, we analysed the double immunofluorescence stained sections with a confocal microscope. In the co-localization studies, sections were studied with a spectral confocal microscope (Leica TCS-SP2-AOBS) at ×63 with an oil-immersion objective and imaging software (Leica Confocal Software Lite Version). All analyses were carried out in sequential scanning mode (lasers 488 Ar, 561 DPSS and 633 He/Ne) to eliminate the possibility of cross-bleeding between channels. Results were obtained from projections in the *z*-axis.

### Prolyl oligopeptidase enzymatic activity

Serum or microdialysate samples (50 μl) were used to assay PREP activity by measuring the fluorescence released from the substrate N-carbobenzoxy-glycyl-prolyl-7-amido-4-methyl-coumarin (Z-Gly-Pro-AMC, 200 μM), as previously reported [17], and in the absence or presence of 50 nM KYP-2047. The activity sensitive to the inhibitor is reported as PREP activity.

### Western blotting

Rat tissues were homogenized in 50 mM phosphate buffer pH = 7 (5 volumes), and further disrupted by sonication (2 × 5 s). After a low-speed centrifugation (1000×*g*), samples obtained this way, or human/rat plasma samples (for preparation see below), were diluted 1:1 with loading buffer (100 mm Tris/HCl, pH 6.8, 70 % glycerol, 2 % SDS, 0.005 % bromophenol blue, 10 mM mercaptoethanol) and separated on 10 % polyacrylamide/bis-acrylamide Tris/HCl discontinuous gels. Gels were transferred to nitrocellulose for blotting. Western blot was performed under standard conditions using affinity purified chicken anti-PREP IgY [40], diluted 1:500 with 0.5 m NaCl, 20 mm Tris–HCl pH 7.5, 5 % skim milk and 0.05 % Tween 20 (TTBS) overnight. After that, first antibody was washed three times with TTBS and incubated 1 h with anti-chicken horseradish peroxidase complex (Santa Cruz Biotechnology Inc., Dallas, TX, USA) diluted 1:3000 in TTBS. After washing, protein visualization was performed using a chemoluminescent substrate kit (Pierce, Rockford, IL, USA), following the manufacturer’s instructions. Western blot images were analysed, and the optical density (OD) values of protein bands were determined by using QuantityOne-software (version 4.6.9, Bio-Rad).

### Ibuprofen treatment of PCS rats

Treatment with ibuprofen was performed as performed in Cauli et al. [39]. Rats subjected to portacaval anastomosis, and also those sham operated, were treated daily with S-(1) Ibuprofen (Fluka, Seelze, Germany). The treatment with ibuprofen started 10 days after PCS surgery, or sham operation. Ibuprofen dissolved in sterile saline was injected intraperitoneally. Rats received 0, 5, 15 or 30 mg/kg ibuprofen per day in 0.5 ml/100 g body weight. Rats were sacrificed after 5 weeks of treatment.

### Patients with liver cirrhosis and controls

Forty patients with diagnosed liver cirrhosis after histological study, and 20 healthy controls without no clinical, analytical, serologic and ecographic evidence of liver malfunction, were enrolled in the study after informed consent. After performing the psychometric tests, patients were divided into those diagnosed with or without MHE (see below). The composition of the groups, the number of subjects, age, sex and aetiology of the liver disease are given in Table 1.

**Table 1:** Demography of controls and patients

	Control	Patients without MHE	Patients with MHE
Total individuals (male/female)	20 (8/12)	20 (15/5)	20 (12/8)
Age^a^ (years)	44 ± 11	55 ± 10	65 ± 10
Alcohol	–	20	20
Child-Pugh A/B/C	–	13/7/0	12/8/0
MELD score^a^	–	10 ± 3	9.8 ± 3
Ascites	–	1	2

*MHE* minimal hepatic encephalopathy, *MELD* model end-stage liver disease

^a^mean ± SD

The study protocol complied with the ethical guidelines of the Declaration of Helsinki of 1975 and was approved by the Scientific and Ethical Committees of the Hospital.

### Diagnosis of minimal hepatic encephalopathy

MHE was diagnosed using the Psychometric Hepatic Encephalopathy Score (PHES), recommended as the “gold standard” [4, 32]. PHES comprises five psychometric tests: digit symbol test (DST), number connection test A (NCT-A), number connection test B (NCT-B), serial dotting test (SD) and the line tracing test (LDT). The score in each test and the PHES were calculated adjusting for age and education level by means of Spanish normality tables (www.redeh.org). Patients were classified as having MHE when the score was less than −4 points [8].

### Collection of plasma and serum

Blood (5 ml) was taken in BD Vacutainer tubes with or without EDTA (for plasma and serum, respectively) and centrifuged at 500×*g* for 10 min. The supernatant was collected and stored frozen at −80 °C in aliquots of 500 μl.

### Determination of nitrates + nitrites

Nitrate (NO_3_^−^) was measured in plasma as nitrite after enzymatic conversion by nitrate reductase as previously described [6, 39].

### Determination of ammonia

Ammonia content in human blood was measured as described previously [5]. By duplicate, aliquots of blood (150 μl) were added to 150 μl of ice-cold 10 % trichloroacetic acid. After centrifugation at 12,000×*g* for 10 min at 4 °C, the supernatant was collected and neutralized with 15 μl of KHCO_3_ to pH 7. In duplicates, 50-μl samples were mixed with 32.5 μl of the reaction mixture (phosphate buffer 0.2 M, pH 8; α-ketoglutarate 0.6 M pH 7; NADH 10 mM) and added 10 μl of glutamate dehydrogenase. Ammonia was measured with a microplate fluorimeter (Fluoroskan Ascent, Thermoscientific) using excitation and emission filters of 355 and 460 nm, respectively. Ammonia from PCA rats was measured using the kit II Ammonia Arkray test (PocketChem BA, Arkray) according the kit protocol, using 20 μL of blood.

### Isolation of lymphocytes and activation of soluble guanylate cyclase by the NO-generating agent S-nitroso-N-acetylpenicillamine (SNAP) in intact lymphocytes

Lymphocytes were obtained as previously described [40, 41] and resuspended in 800 μl Locke’s solution without magnesium (in mM: NaCl, 154; KCl, 5.6; NaHCO_3_, 3.6; CaCl_2_, 2.3; glucose, 5.6; 4(2-hydroxyethyl)-1-piperazine-ethanosulfonic acid, 5; pH 7.4) containing 0.3 mM 3-isobuthyl-1-methylxantine (IBMX, Sigma, Germany). Quality of the preparation was assessed by microscopy, and the viability was determined by trypan blue assay. Lymphocytes were distributed in 200 μl aliquots and incubated for 15 min at 37 °C in a water bath before addition of SNAP (1 mM, Molecular Probes, Eugene, OR, USA). Incubation was continued for 15 min. Subsequently, the cells were collected by centrifugation at 450×*g* for 10 min. The pellet was resuspended in 200 μl of lysis solution (0.5 % dodecyl-trimethylammonium bromide). Protein was measured by the BCA™ Protein Assay (Thermo Scientific, Rockford, IL, USA).

### Determination of cGMP in plasma and lymphocytes

The level of cGMP in plasma and in lymphocytes was determined using the BIOTRAK™ cGMP enzyme immunoassay kit from Amersham (GE Healthcare, Life Sciences, UK).

### Determination of interleukins in serum

IL-6 was determined using the BIOTRAK Easy ELISA system from Amersham (GE Healthcare, Life Sciences, UK); IL-18 was measured using an ELISA system from Bender MedSystems GMbH (Austria). The measurements were performed with a micro plate reader (Multiskan Ascent, Thermo Electron Corp.).

### Statistical analysis

Values are given as mean ± standard deviation. We used *t* test to analyse immunohistochemical or western band densities, comparing the results with the control values for every tissue. Enzymatic activity results on serum samples were analysed by one-way ANOVA followed by post hoc Bonferroni test. Calculations were performed using GraphPad PRISM Version 4.0. The probability level accepted for significance was *p* < 0.05. Correlation analysis was performed using the SPSS software, Version 17.0 (SPSS Inc, Chicago, USA), and two-sided *p* values <0.05 were considered significant.

## Results

### PREP changes in the brain of HE rat model

**Fig. 1:**
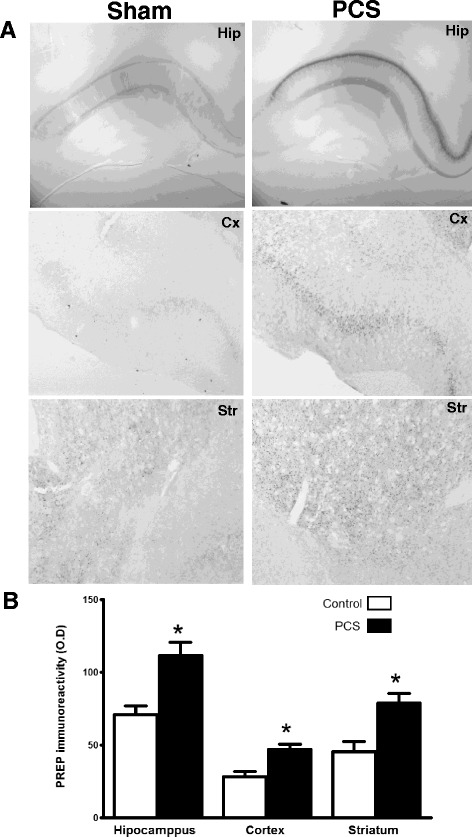
The expression of PREP in the brain of PCS rats is substantially increased as shown by **a** immunohistochemistry of sections of the hippocampus (*Hip*), frontal cortex (*Cx*) and striatum (*Str*), compared with sham operated rat brain. This increase in density was quantitated as represented in (**b**) (*n* = 4–5)

**Fig. 2:**
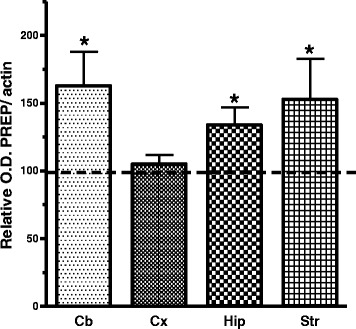
PREP expression was also detected increased by western blotting in the brain of PCS rats, compared with sham operated control rats (line at 100 %), especially in the cerebellum (*Cb*, *n* = 9), hippocampus (*Hip*, *n* = 6) and striatum (*Str*, *n* = 4). The increase was not significant in the cortex (*Cx*, *n* = 4)

After surgically subjected to portacaval shunt (PCS), rats develop liver atrophy, hyperammonemia, inflammation and minimal hepatic encephalopathy similarly to the situation in patients with liver cirrhosis [5]. Accordingly, the brain of PCS rats was analysed for PREP levels by immunohistochemistry and western blot. Figure 1a shows PREP immunohistochemistry of different areas of the PCS rat brain after 6–12 weeks and compared with the images of the same areas of the brain from sham rats. There was a significant increase of PREP immunostaining in the PCS model. The increase was especially marked in the CA1 layer of the hippocampus. The increase ranged from 40 to 100 % depending on the brain area. The optical density difference for the different areas analysed is shown in Fig. 1b. To verify these results, we dissected the cerebellum, cortex, hippocampus and striatum, homogenized them and measured PREP levels by western immunoblotting. These results are shown in the Fig. 2. With the exception of cortex, PREP levels were found increased significantly in all areas of the brain of PCS rats (Fig. 2).

### PREP changes in the plasma of HE rats

**Fig. 3:**
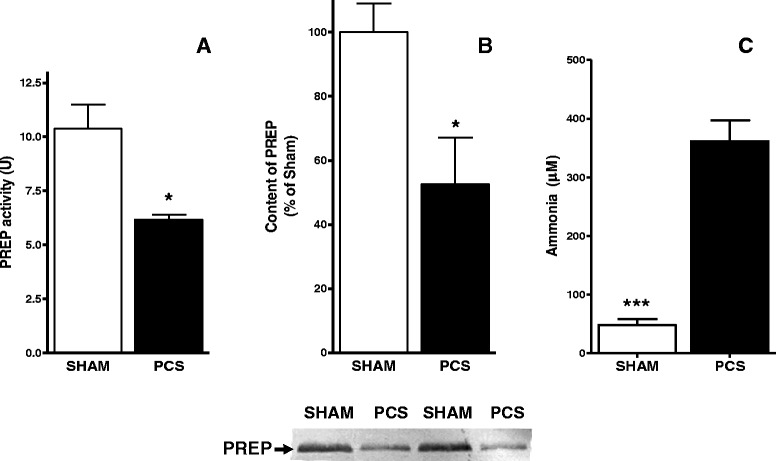
PREP activity (**a**) and protein levels in plasma, determined by western blotting (**b**), are decreased (*p* < 0.05) in PCA rats (*black bar*, *n* = 4) compared with sham operated control rats (*white bar*, *n* = 5). Representative blots are show in the lower panel. **c**, ammonia levels in PCA are increased relative to control

We measured PREP activity and protein levels in plasma from PCS rats (Fig. 3). We found a statistically significant (*p* = 0.023) reduction of PREP activity in PCS (6.167 U ± 0.2333, *n* = 3) compared to sham operated controls (11.97 ± 1.249, *n* = 4). In addition, PREP protein levels in PCS plasma, measured by western blot, were also found decreased to 52.58 % ±14.63, compared to sham operated controls (*p* < 0.05) (Fig. 3b). This is consistent with the drop in PREP activity. The decrease on PREP was parallel to an increase level of ammonia (Fig. 3c). Blood ammonia levels were increased (*p* < 0.001) in PCS rats (361 ± 39 μM) compared with sham operated rats (51 ± 13 μM).

### PREP co-localizes with neurons and glial cells in the brain of PCS rats

**Fig. 4:**
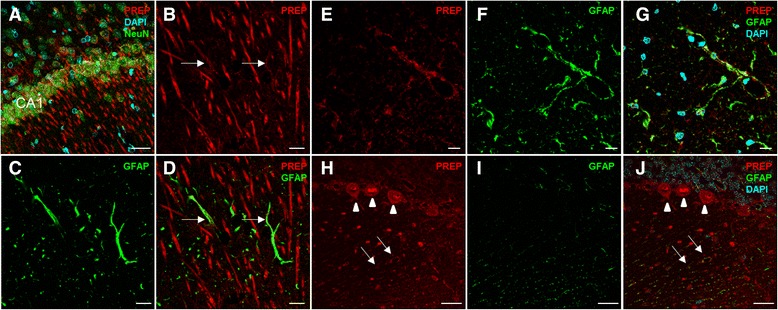
PREP is expressed in neurons and astrocytes in hippocampus and cerebellum of PCS rats. PREP (*red*) is expressed mainly in the neurons of the CA1 region of the hippocampus visualized with NeuN (*green*) (**a**). High magnification images showed co-localization (*arrows*) of PREP (*red*) with astrocytes stained with glial fibrillary acidic protein (GFAP; *green*) in the hippocampus (**b**–**d**), in the white matter of the cerebellum (**e**–**g**) and the cerebellar cortex (**h**–**j**). To note is the strong PREP expression in Purkinje cells (*arrow heads* in (**h**, **j**)) also co-localization with the glia Bergman (*arrows*). Nuclear marker: DAPI (*cyan*). Scale bar: **b**–**g** 10 μm; **a**, **h**–**j** 30 μm

Cellular localization of PREP in different areas of the PCS rat brain was investigated by confocal microscopy (Fig. 4). PREP was clearly positive in neurons, especially in the CA1 layer of the hippocampus (Fig. 4a) and cerebellar Purkinje cells (Fig. 4h) as described before [13, 14]. However, PREP was not found only in neurons, as is normally found in healthy rat brain [13, 14]. In PCS brain, PREP was observed within hippocampal astrocytes located in the molecular layer and in the dendritic region of the neurons of the CA1 layer (Fig. 4d) and remarkably in glial cells of the cerebellar cortex (Fig. 4e) and white matter (Fig. 4g).

### Extracellular PREP is increased in the brain of HE model rats

**Fig. 5:**
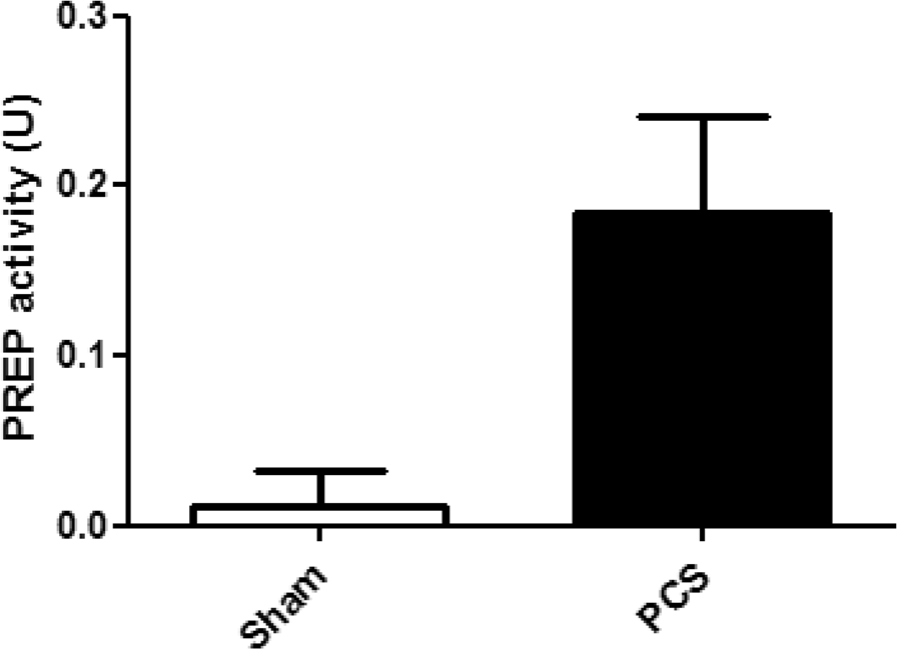
Brain extracellular PREP activity measured in the dialysate of cerebellum microdialysis of sham operated control rats (*white bar*) and PCS rats (*black bar*) (*n* = 4)

We have shown that hyperammonemic and portacaval shunt models of HE in rats develop neuroinflammation in specific areas of the brain, especially in cerebellum [6, 40]. As PREP was increased in the brain of PCS rats, we wanted to test if the peptidase was also being secreted from the cells where this was up-regulated. Accordingly, we collected extracellular fluid from the cerebellum of PCS rats by microdialysis. We found that the amount of extracellular PREP is dramatically increased in PCS rats, compared with the sham operated controls as shown in Fig. 5. The activity detected in sham operated rats was just above the limit of detection (0.012 ± 0.02033 U, *N* = 5), while in PCS rats, PREP activity levels were increased almost 20 times (0.1845 ± 0.05668 U, *N* = 4).

### PREP levels are restored to normal upon ibuprofen treatment of PCS rats

**Fig. 6:**
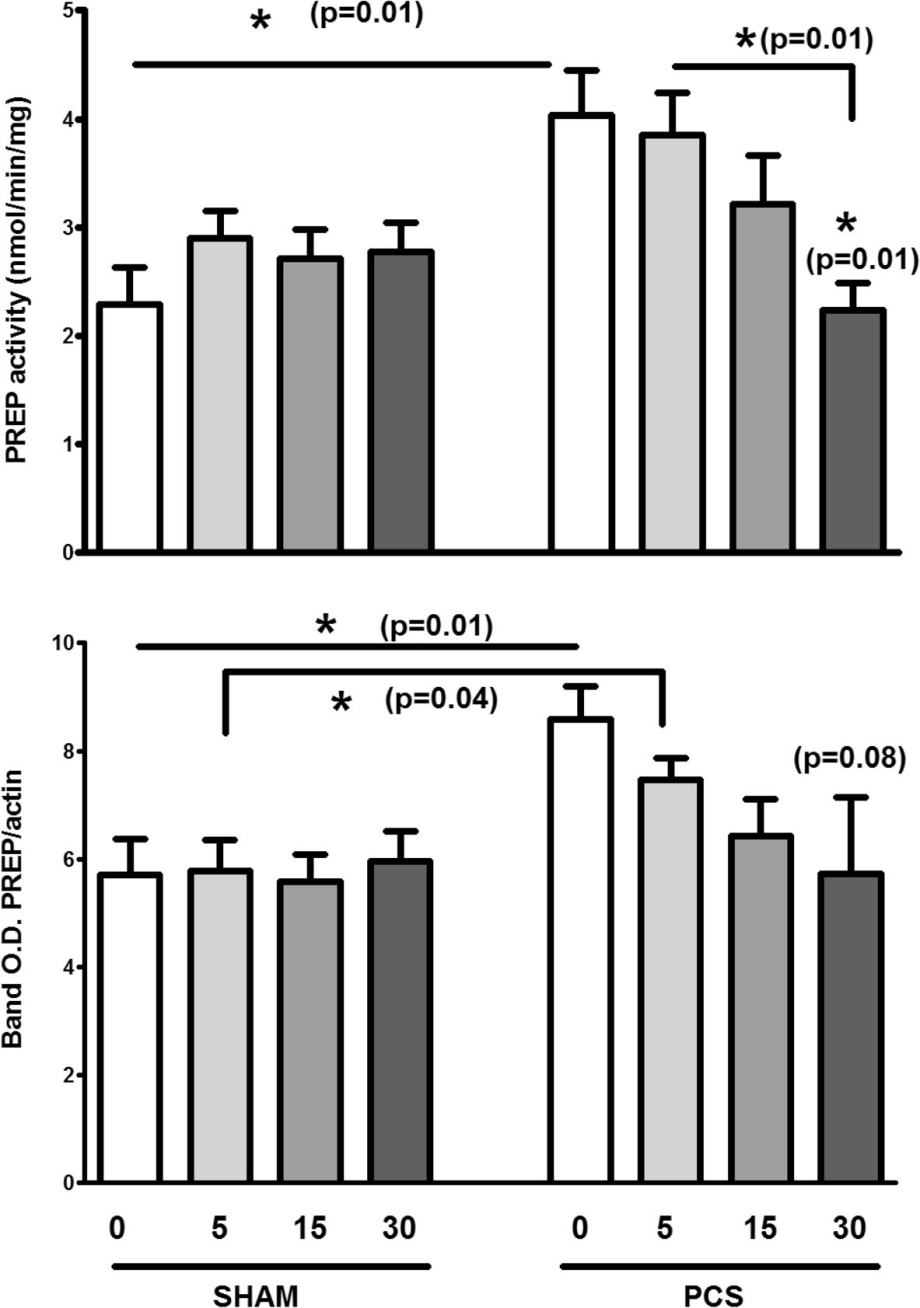
PREP increased in PCA rats is recovered to control level after ibuprofen treatment. Groups of 4–5 animals of control sham operated rats (*left*) and PCA rats (*left*), were administrated with 0, 5, 15 and 30 mg/kg for 4 days, and striatal PREP levels of activity (*upper graph*) and protein (*lower graph*) were measured in both groups. The *shade* of *grey* in the bars increases with the increase on ibuprofen dose as showed. Statistical *p* values between selected bars are shown

We have reported that treatment with an anti-inflammatory agent, ibuprofen, restores motor function in PCS rats and normalizes glutamate in substantia nigra pars reticulata (SNr) [39]. PCS rats, and sham operated, were treated with 0, 5, 15 and 30 mg/kg of ibuprofen for 5 days. Figure 6 shows the levels of PREP activity and protein in striatum of these treated rats. It is observed that striatal PREP is increased in PSC untreated rats compared with sham untreated animals, as shown in Figs. 1 and 2, but a gradual decrease of the levels to control values is achieved as the ibuprofen dose is increased.

### PREP activity is reduced in cirrhotic patients regardless of HE, but PREP protein was found reduced only in patients with MHE

**Fig. 7:**
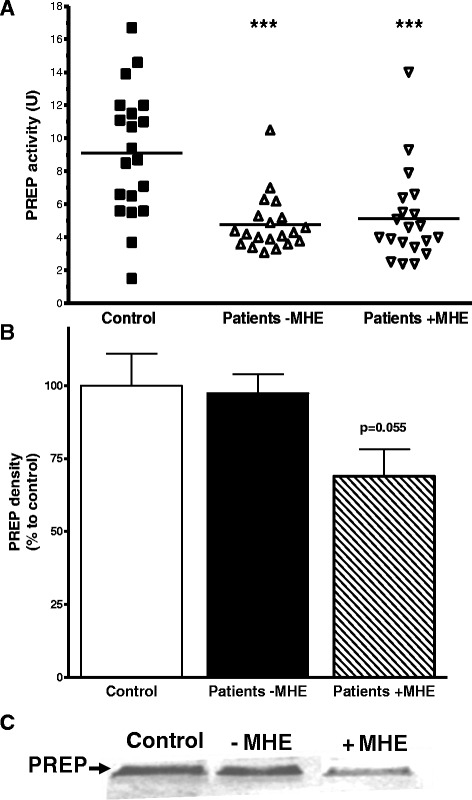
**a** Average PREP activity in cirrhotic patients without (*triangles*) or with (*inverted triangles*) symptoms of MHE was significantly decreased compared with healthy subjects (***p < 0.001). **b** The levels of PREP protein in patients without MHE (*black bar*) showed no significant change, but these levels are apparently reduced in patients with MHE (*hatch pattern bar*), in comparison with PREP levels in control healthy subjects (*white bar*). A representative western blot is shown in **c**

We measured the activity of PREP in plasma from patients with liver cirrhosis, which presented the absence (*n* = 20) or presence (*n* = 20) of the symptoms of MHE and compared with the measurements from healthy volunteers (*n* = 20). PREP activity was found significantly decreased in plasma from all cirrhotic patients (Fig. 7a). Relative to control, cirrhotic patients showed a decrease of 65 % (patients without MHE, *P* < 0.001) and 50 % (patients with MHE, *p* < 0.001). However, the difference between patients with or without MHE was not significant.

On the other hand, when the PREP protein levels were measured by western blot (Fig. 7b), no change was observed in PREP protein in cirrhotic patients without MHE, but it was found somehow reduced in patients presenting MHE.

### PREP activity correlates with IL-6, IL-18 and GMPc levels in cirrhosis

It is known that systemic inflammation, upon liver failure, contributes to encephalopathy in cirrhosis, in a process that is characterized by microglial activation with the increase of cytokines and activation of GMPc-pathways [42–44]. Several of those parameters, and others that are commonly measured in clinical samples over diagnosis of cirrhosis and/or MHE, were assayed in the patient cohort in this study to confirm the assessment of diagnosis and to correlate them with those values of PREP activity found modified. The results are shown in Table 2 along with the PHES. The changes found in nitrates, plasma cGMP, basal cGMP in lymphocytes and SNAP-induced cGMP increase, as well as IL-6, IL-18 and ammonia, were consistent with described changes in cirrhosis. Creatinine, bilirubin, albumin and INR are clinical parameters frequently utilized in cirrhotic patients to estimate MELD and Child-Pugh. We observed that we confirmed that bilirubin and albumin were significantly modified in cirrhotic patients. It was also observed that the changes in cGMP, IL-6 and IL-18 correlated with MHE. When we compared the parameters measured with the changes found in the levels of PREP, we found that the changes in IL-6, IL-18, both plasma and lymphocyte basal cGMP and SNAP-induced cGMP, as well as total bilirubin and albumin, correlated with PREP changes (Table 3). To evaluate if the changes in peptidase levels correlated with the severity of the pathologic state of the patients, we compared those results with the scores obtained by Child-Pugh or the model end-stage liver disease (MELD). We did observe a strong correlation between Child-Pugh and MELD scores and the PREP levels (Table 3). In further analysis, no correlation was found between PREP activity and performance in different coordination and attention tests, such as Stroop, map search or bimanual coordination test (not shown).

**Table 2:** Values of parameters in the different groups of patients with liver disease and in controls

Parameter	Controls	Patients without MHE (*p* vs. control)	Patients with MHE (*p* vs. control)	*p* values (without MHE vs. with MHE)	Global ANOVA
					*p* values
PHES	−0.2 ± 0.8^a^	−0.4 ± 0.8	−6 ± 2	<0.001	<0.0001
			*p* < 0.001		
Nitrates + Nitrites (μM)	18 ± 3	24 ± 8	29 ± 8	ns	<0.0001
		*p* < 0.05	*p* < 0.001		
cGMP in Plasma (pmoles/ml)	4 ± 1	7 ± 3	10 ± 4	<0.05	<0.0001
		*p* < 0.05	*p* < 0.001		
Lymphocyte basal cGMP (pmol/mg prot)	0.169 ± 0.06	0.078 ± 0.06	0.047 ± 0.02	ns	<0.0001
		*p* < 0.001	*p* < 0.01		
SNAP-induced cGMP increase (fold)	11 ± 3	21 ± 9	22 ± 11	ns	0.0004
		*p* < 0.01	*p* < 0.01		
ANP (ng/ml)	0.014 ± 0.003	0.015 ± 0.002	0.015 ± 0.002	ns	ns
IL-6 (pg/ml)	1 ± 0.6	2.5 ± 1.5	4.7 ± 2.1	<0.001	<0.0001
		*p* < 0.05	*p* < 0.001		
IL-18 (pg/ml)	174 ± 80	281 ± 138	388 ± 138	<0.05	<0.0001
		*p* < 0.05	*p* < 0.001		
Creatinine (mg/dl)	0.74 ± 0.12	0.77 ± 0.19	0.86 ± 0.45	ns	ns
Total bilirubin (mg/dl)	0.61 ± 0.16	1.42 ± 0.94	1.05 ± 0.83	ns	0.005
		*p* = 0.003			
Albumin (g/dl)	4.49 ± 0.14	3.90 ± 0.56	3.70 ± 0.59	ns	<0.001
		*p* = 0.001	*p* < 0.001		
INR	1.06 ± 0.05	1.22 ± 0.36	1.18 ± 0.17	ns	0.08
Ammonia (μM)	72 ± 22	127 ± 40	131 ± 35	ns	<0.001
		*p* < 0.001	*p* < 0.001		

Differences between groups were assessed with ANOVA plus post hoc Tukey test

*MHE* minimal hepatic encephalopathy, *PHES* Psychometric Hepatic Encephalopathy Score, *ANP* atrial natriuretic peptide, *IL-6* interleukin-6, *IL-18* interleukin-18

^a^Values are expressed as the mean ± SD

**Table 3:** Correlations of the parameters studied with PREP levels in plasma of cirrhosis and control subjects

Parameter	Correlation statistical parameters
PHES	*r* = ns
	*p* = ns
Child-Pugh	*r* = −0.484
	*p* = <0.001
MELD	*r* = −0.377
	*p* = 0.004
Creatinine	*r* = ns
	*p* = ns
Total bilirubin	*r* = −0.281
	*p* = 0.031
Albumin	*r* = 0.362
	*p* = 0.007
INR	*r* = ns
	*p* = ns
IL-6	*r* = −0.467
	*p* = 0.001
IL-18	*r* = −0.295
	*p* = 0.039
cGMP plasma	*r* = −0.333
	*p* = 0.019
Lymphocyte basal cGMP	*r* = 0.431
	*p* = 0.003
SNAP-induced cGMP	*r* = −0.413
	*p* = 0.003
Nitrates + nitrites	*r* = ns
	*p* = ns
ANP	*r* = ns
	*p* = ns
Ammonia	*r* = ns
	*p* = ns

*r*Pearson correlation coefficient, *PREP* prolyl oligopeptidase, *FAP* fibroblast associated protein, *DTT* dithiothreitol, *PHES* Psychometric Hepatic Encephalopathy Score, *IL-6* interleukin-6, *IL-18* interleukin-18, *ANP* atrial natriuretic peptide, *MELD* model end-stage liver disease, *ns* not significant

## Discussion

Recently, PREP has been proposed as a modulator of the inflammatory response in different pathologies of the lung [29, 30, 45–49]. This response involves a multistep process resulting in the formation of the chemoattractant peptide, N-acetylated-proline-glycine-proline (Ac-PGP) a biomarker for chronic obstructive pulmonary disease (COPD) [29]. Furthermore, it has been shown that PREP is involved in the generation of N-acetyl-seryl-aspartyl-lysyl-proline (Ac-SDKP) form thymosin-β_10_ in the kidney [50, 51] and that these peptide levels decrease in renal fibrosis and inflammatory cell infiltration in hypertensive rats [52]. Moreover, PREP has been reported overexpressed in inflammatory cells in culture [28] and in brain glial cells upon neuroinflammatory insults [20]. On the other hand, plasma PREP has been reported particularly reduced in patients with relapsing remitting and primary progressive-multiple sclerosis, pathologies with a prominent inflammatory component [19, 37]. In this paper, we measured inflammatory markers in cirrhotic patients and correlated the circulating levels of PREP in search for a relation to neuroinflammation developed in MHE and compare these correlations with PREP expression in a model of MHE in rats.

We found that circulating PREP activity was substantially reduced in all cirrhotic patients but those changes did not have evident correlation to MHE. However, when assaying PREP protein by western blot, a decrease was only found in patients with MHE. It is important to note, that it has been established that PREP activity levels do not necessary correspond to protein levels and that PREP has also non-catalytic functions [12, 13, 16, 24]. Thus, PREP activity levels detected in plasma are the result not only of the levels of protein but also of the levels of activity modulators, like reactive oxygen species, α-2-macroglobulin and other peptides [19]. We have recently shown that cirrhotic patients, with or without MHE, show increased oxidative stress in blood compared with control subjects, as reflected by an increased lipid peroxidation, DNA oxidation, protein carbonylation, high 3-nitrotyrosine level and higher oxidized/reduced glutathione ratio [53]. It is therefore likely that increased oxidative stress may contribute to reduced PREP activity in cirrhotic patients.

On the other hand, it has been described that MHE correlates with increased levels of the pro-inflammatory cytokines IL-6 and IL-18 and with altered nitric oxide (NO)-cGMP homeostasis and increased activation of soluble guanylate cyclase by NO in freshly isolated lymphocytes [8, 40]. Specially, increased levels of serum IL-6 and IL-18 correlated with cognitive impairment in patients with cirrhosis of the liver and minimal hepatic encephalopathy [8]. We found significant differences in the levels of plasma cGMP, IL-6, and IL-18, between patients without MHE relative to those who presented MHE (Table 2). In further correlations, we found that these particular parameters, plasma cGMP, IL-6 and IL-18, did significantly correlate with PREP activity. In addition, parameters like lymphocyte basal cGMP, SNAP-induced cGMP, bilirubin and albumin, that are markers of cirrhosis in general, and some of them are parameters to assay disease severity, remarkably also correlated with the general PREP decrease in cirrhosis (Table 3). Inversely, levels of ammonia, as expected, were significantly increased in cirrhosis, but they did not correlate with the changes in PREP. This, and the correlations found with plasma cGMP and IL’s, might be an indication that PREP control is mechanistically connected to the changes caused by increased in ammonia, rather than with increase of ammonia itself. It is interesting to note that we found here a negative correlation between PREP activity and the severity of cirrhosis, as Child-Pugh and MELD scores. Child-Pugh assigns a score from 1 to 3 reflecting the severity of ascites, hepatic encephalopathy, international normalized ratio (INR) of prothrombin time, albumin and bilirubin. On the other hand, the scale for MELD system is wider (from 0 to 50) and based on serum bilirubin, serum creatinine and prothrombin time, which are more objective variables in estimating mortality in patients with the chronic disease [54]. We found here that circulating levels of total bilirubin, and also those of albumin, correlated also with the changes in PREP activity (Table 3). In general, the greater the score in both Child-Pugh and MELD systems, the greater the severity of the liver disease, and the lower the circulating PREP activity level, according to our results. However, this observation has to be taken with some caution, since our study did not include cirrhosis patients with a Child-Pugh C score, mainly because of the complex clinics of the disease at this stage.

Animal models of HE have been established and characterized. Upon portacaval shunt (PCS), or hyperammonemia (HA), conditions that mimic liver failure, rats develop encephalopathy [5, 6, 55, 56]. In this work, we found that PREP immunoreactivity was increased in the hippocampus, cortex and cerebellum in the PCS model. The PREP increase was also confirmed by western blotting for the cerebellum, striatum and hippocampus. In addition, we recorded an increase of PREP localized in astrocytes in the hippocampus and cerebellum. Conversely, but in parallel with the results found in cirrhotic patients, we also detected a decrease in circulating PREP in PCS rats. The alterations in circulating levels of PREP in PCS rats, measured enzymatically and by western, are in parallel with those found in cirrhotic patients with MHE; the activity of PREP is reduced in plasma of cirrhotic patients with or without MHE, but the amount of protein is only lower in plasma of cirrhotic patients with MHE, with respect to the control, as well this is reduced in PCS rats. This supports that PCS rats are a good model reproducing the alterations in PREP found in patients with MHE.

Additionally, we measured extracellular PREP in the cerebellum in PCS rats and found that its activity is substantially increased. To our knowledge, this is the first time extracellular PREP has been detected in vivo in the brain. These results may indicate that concomitant to central inflammation there is an up-regulation of PREP in the brain and this might be translocated to the extracellular space, at least in the cerebellum. The strong increase in the extracellular PREP activity in rats with HE that we observed may have important consequences in the modulation of neurotransmission. In fact, the finding that ibuprofen restores PREP levels to normal in PCS rats, further supports PREP involvement in the inflammatory processes. Several neuropeptides which have roles in neuroinflammation are substrates of PREP, such as neurotensin, substance P, TRH and neuropeptide Y, among others [11]. The increase in extracellular PREP activity in HE may lead to enhanced degradation of these substrates, reducing their extracellular levels, at least in cerebellum. This could result in altered neurotransmission which, in turn, would contribute to the cognitive and motor alterations found in rats with HE. It is important to note that there have been relatively large number of studies measuring changes in neuropeptides in response to PREP inhibition and there has not been consensus on a true correlation of PREP activity and peptide levels (data reviewed in [10, 11, 20]). These divergences might be due to the fact that experimental paradigms tested have not considered the inflammatory factors or glial activation. In fact, the lack of correlation between neuropeptide levels and PREP activity in several experiments has been explained by the inaccessibility of PREP, putatively located intracellular, to cleave the neuropeptides, located in the extracellular milieu [26, 27].

The mechanism by which HE increases extracellular activity of PREP remains unknown. A possible explanation would be that neuroinflammation may result in enhanced release to the extracellular space of PREP, which could be released either from neurons or from astrocytes. In normal rats, PREP is present mainly in neurons [13, 14]. However, we show here that in PCS rats it is also expressed in glial cells. This suggests that neuroinflammation induces the expression of PREP in glial cells and its release to the extracellular fluid. This would be in agreement with previous studies showing that activated microglia can express and secrete PREP [20, 22], which, in vitro, is toxic for neurons [28]. In HE, as mentioned above, the increase in extracellular PREP may contribute to alter neuronal function and neurotransmission, contributing to the neurological alterations in MHE. This is strongly supported by the fact that ibuprofen treatment of PCS rats restores the levels of PREP and, as reported before, also restores the levels of glutamate in SNr and improves the motor performance of the rats [5, 39]. These effects have also been observed in other models of hepatic encephalopathy as bile duct ligation as well as in hyperammonemia [5, 6].

Recently, it has been proposed a synergistic contribution of inflammation and hyperammonemia to HE [7, 8, 42, 57, 58]. On the other hand, changes of PREP are apparently correlated to systemic and central inflammation. We observed in PCS rats an increase of PREP in brain areas, but a decrease of circulating PREP activity. Although, and due to the techniques available to detect PREP, this cannot be measured in the brain of patients, we assume that PREP increase in brain is also occurring in cirrhosis. The molecular mechanisms of these changes are still unaccounted; however, our data and previous studies indicate that there is a connection with inflammatory signalling, operating through different routes in the brain and in circulation. In the brain, the PREP up-regulation might be part of the integral process of glial activation, and most probably it is intervening on the modulation of the extracellular matrix upon inflammation. The information is still scarce to reach any conclusion yet for the changes of PREP in circulation. The circulating levels of PREP activity are accounted not only by its protein levels but also by endogenous modulators and oxidative conditions. The relative levels of circulating PREP protein are very low, around the limits of detection of western blotting technique. Thus, our assay of protein levels with this technique has to be taken with care. On the other hand, we have shown before that PREP activity changes might be due to an increase of an endogenous PREP inhibitor, α2-macroglobulin (α2M), other circulating peptides not yet identified, and on reactive oxygen species (ROS), since PREP is particularly sensitive to oxidative conditions [19]. We have shown that oxidative stress is increased in blood of cirrhotic patients [53], which may reduce PREP activity. However, we found no relation of PREP activity to the levels of α2M or effect of reducing agents, like DTT, on enzymatic activity in multiple sclerosis samples or in this study (data not shown).

## Conclusions

In this paper, we report that PREP level is increased in the brain of HE models, and the peptidase is, at least in the cerebellum, secreted out from the cells. PREP secretion has only been seen in vitro in T cells [28]. Furthermore, circulating PREP activity was observed substantially depressed in cirrhosis and in PCS rats with MHE. Low circulating PREP activity has been observed upon neuroinflammation in multiple sclerosis [19]. Our findings did not show a clear correlation of PREP activity decrease in circulation with HE. However, a relationship might be present, but it is masked due to the presence of systemic inflammation, which in turn might be also affecting PREP activity levels. This idea is supported by the fact that markers of MHE in cirrhosis correlated with PREP changes. On the other hand, PREP might indeed be directly related to neuroinflammation as we observed a restoration of PREP levels upon administration of the anti-inflammatory agent ibuprofen to PSC rats. These results are encouraging and open research lines to further study PREP and its relation with the aetiology of inflammatory conditions, aiming in part to determine if PREP might be considered a valuable biomarker for inflammation.
